# Behavior of Eosinophil Counts in Recovered and Deceased COVID-19 Patients over the Course of the Disease

**DOI:** 10.3390/v13091675

**Published:** 2021-08-24

**Authors:** Ricarda Cortés-Vieyra, Sergio Gutiérrez-Castellanos, Cleto Álvarez-Aguilar, Víctor Manuel Baizabal-Aguirre, Rosa Elvira Nuñez-Anita, Angélica Georgina Rocha-López, Anel Gómez-García

**Affiliations:** 1 Centro de Investigación Biomédica de Michoacán, División de Investigación Clínica, Instituto Mexicano del Seguro Social (IMSS), Morelia 58341, Michoacán, Mexico; sergio.gutierrezc@imss.gob.mx; 2 División de Estudios de Posgrado, Facultad de Ciencias Médicas y Biológicas “Dr. Ignacio Chávez”, Universidad Michoacana de San Nicolás de Hidalgo (UMSNH), Morelia 58020, Michoacán, Mexico; cleto.alvarez@imss.gob.mx (C.Á.-A.); ginrocha@gmail.com (A.G.R.-L.); 3 Coordinación Auxiliar Médica de Investigación en Salud, IMSS, Morelia 58000, Michoacán, Mexico; 4 Centro Multidisciplinario de Estudios en Biotecnología, Facultad de Medicina Veterinaria y Zootecnia, UMSNH, Morelia 58890, Michoacán, Mexico; victor.baizabal@umich.mx (V.M.B.-A.); qfbrena@hotmail.com (R.E.N.-A.)

**Keywords:** eosinophils, neutrophils, white blood cell differential counts, neutrophil-to-lymphocyte ratio, COVID-19

## Abstract

Knowledge about the immune responses against severe acute respiratory syndrome coronavirus 2 (SARS-CoV-2) infection, particularly regarding the function of eosinophils, has been steadily emerging recently. There exists controversy regarding the implications of eosinophils in the coronavirus disease 2019 (COVID-19)’s pathology. We report a retrospective cohort study including the comparison of leukocyte counts in COVID-19 patients, considering the outcomes of recovery (*n* = 59) and death (*n* = 60). Among the different types of leukocytes, the eosinophil counts were those that showed the greatest difference between recovered and deceased patients. Eosinopenia (eosinophil count < 0.01 × 10^9^/L) was more frequently observed in deceased than recovered patients (*p* = 0.0012). The eosinophil counts more rapidly increased and showed a greater proportion over the course of the disease in the recovered than deceased patients. Furthermore, the estimated survival rate was greater in patients without eosinopenia than in patients with eosinopenia (*p* = 0.0070) during hospitalization. Importantly, recovered but not deceased patients showed high negative correlations of the eosinophils with the neutrophil-to-lymphocyte ratio (NLR) and neutrophil counts at Day 9 of the onset of clinical symptoms (*p* ≤ 0.0220). Our analysis suggests that eosinopenia may be associated with unfavorable disease outcomes and that the eosinophils have a beneficial function in COVID-19 patients, probably contributing by controlling the exacerbated inflammation induced by neutrophils.

## 1. Introduction

In December 2019, an outbreak of pneumonia of unknown etiology was reported in Wuhan, China. On 11 February 2020, the World Health Organization (WHO) designated the name “COVID-19” to the disease caused by “severe acute respiratory syndrome coronavirus 2” (SARS-CoV-2), identified in 2019 [1]. Since then, the global spread of the virus has been rapid, infecting more than 172 million people and causing more than 3 million deaths by June 2021, according to the Johns Hopkins Coronavirus Resource Center [2].

Although several research groups have reported many clinical anomalies associated with the unfavorable progression of COVID-19, such as increases in lactate dehydrogenase, alanine aminotransferase, aspartate aminotransferase, bilirubin, creatinine, cardiac troponin, D-dimers, procalcitonin, and C-reactive protein (CRP) [3,4,5,6], a high neutrophil-to-lymphocyte ratio (NLR) has received special attention [5,7,8]. The behavior of eosinophils in a state of eosinopenia (i.e., a reduction in circulating eosinophils < 0.01 × 10^9^/L) [9], as has previously been described in response to systemic inflammation [10,11], has also been highlighted in COVID-19 [3,12,13,14,15,16,17].

Eosinophils are leukocytes involved in allergic diseases and host protection against parasites through the release of cytokines/chemokines, mediators, antimicrobial peptides, eosinophil extracellular traps (EETs), and cytotoxic granule cationic proteins, such as major basic protein (MBP), eosinophil peroxidase (EPO), eosinophil cationic protein (ECP), and eosinophil-derived neurotoxin (EDN) (7–9). In addition to the proinflammatory effects of eosinophils, evidence in mice has indicated that eosinophils play important roles as regulatory cells involved in protective immunity and antiviral responses [18,19,20,21,22]. 

Eosinopenia has been indicated for the prediction of poor prognosis in COVID-19 [3,12,13,14,15,16,17,23,24]. Additionally, it has been observed that an increase in eosinophils over the course of the disease from initially low levels could be a positive indicator of clinical improvement [25,26]. By contrast, it has been indicated that current studies do not support the use of eosinopenia for the diagnosis of COVID-19 [27]. It has also been suggested that eosinopenia may not be associated with an unfavorable progression of COVID-19 [28] and that eosinopenia may show more diagnostic—and, eventually, prognostic—value than real participation in COVID-19’s pathology [19]. Therefore, the role of eosinophils in COVID-19 remains controversial. Important unresolved issues concerning eosinophils that may affect clinical decisions are (1) whether eosinopenia in COVID-19 patients is associated with the outcome of death and (2) what roles eosinophils play in the development of COVID-19.

The purpose of this research was to understand the role of eosinophils in the progression of SARS-CoV-2 infection. Our analysis suggests that eosinopenia may be associated with unfavorable disease outcomes and suggests a probable inflammation-regulatory role for eosinophils.

## 2. Materials and Methods

### 2.1. Clinical Study 

This retrospective cohort observational study was carried out with patients of the Regional General Hospital l (HGR-1) and the General Hospital of Area 83 (HGZ-83) located at Charo and Morelia, Michoacán, México, respectively. The two medical units, HGR-1 and HGZ-83, are part of the Instituto Mexicano del Seguro Social (IMSS), which provides medical services to approximately 713,600 inhabitants in Morelia.

### 2.2. Laboratory Information 

Venous blood samples were collected in dipotassium ethylenediamine-tetra-acetic acid tubes. All the white blood cell (WBC) analyses were performed with an automatic hematology analyzer (Sysmex XN-2000^TM^, Chuo-Ku, Kobe, Japan). The NLR was obtained by dividing the neutrophil count by the lymphocyte count. The WBC counts and WBC differential counts for eosinophils, neutrophils, basophils, lymphocytes, and monocytes are expressed in absolute values. To determine if there was a difference in the immune responses of COVID-19 patients with respect to those of healthy subjects, the mean values of the NLR and WBC differential counts were compared to the values reported for healthy subjects [29,30].

### 2.3. Ethical Considerations 

The study was authorized by the Ethical Medical Committee of the hospitals included: HGR 1 and HGZ 83.

### 2.4. Statistical Analysis 

Graphical abstracts were created using the Mind the GRAPH^®^ software version 2021. The Kaplan–Meier hazard functions and correlations between eosinophils and NLR and the rest of the WBC differential counts were determined using the IBM^®^ SPSS^®^ Statistics software version 23.0. To compare the NLR and WBC differential counts of COVID-19 patients for different outcomes (recovered vs. deceased), the GraphPad Prism software, version 9.0 (GraphPad Software Inc., ©2020), was used, using the Wilcoxon matched-pairs signed-rank test. Categorical variables are presented as frequency rates and percentages, which were also analyzed with GraphPad Prism, using χ^2^ or Fisher’s tests. The Kaplan–Meier hazard functions were calculated with 95% confidence intervals. The tests for comparisons and correlations with *p*-values less than 0.05 were considered statistically significant.

## 3. Results

### 3.1. Study Development

**Figure 1:**
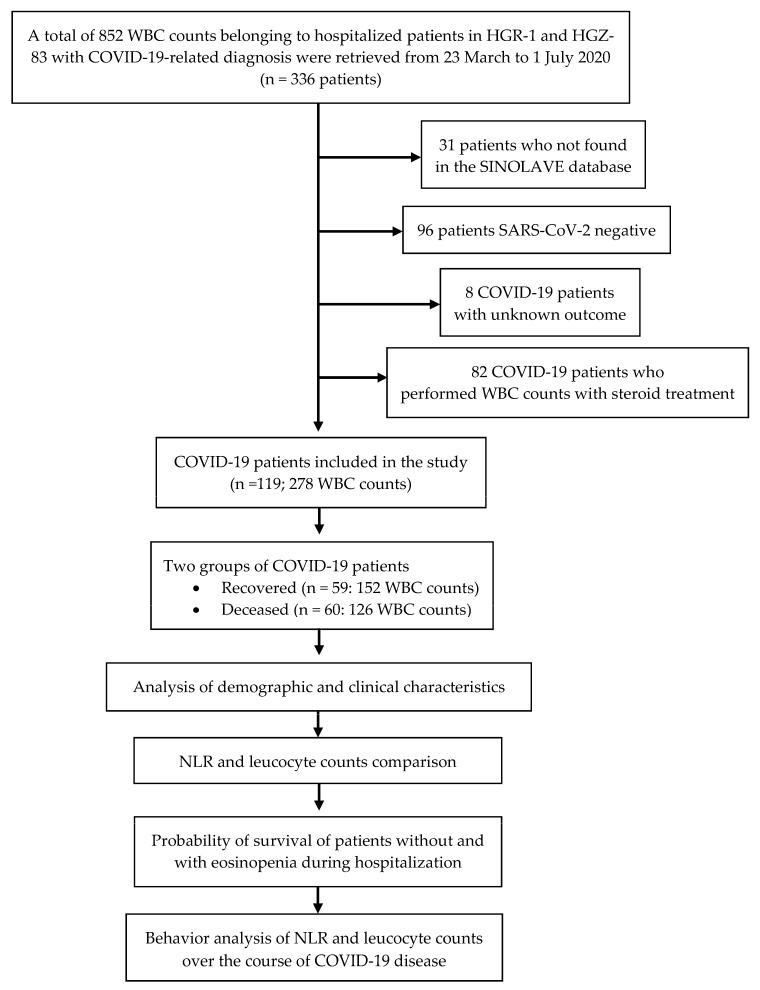
Study flowchart. white blood cell (WBC); Regional General Hospital l (HGR-1); General Hospital of Area 83 (HGZ-83); online notification system for epidemiological surveillance (SINOLAVE); neutrophil-to-lymphocyte ratio (NLR).

A total of 852 white blood cell WBC counts from 336 patients admitted to HGR 1 and HGZ 83 from 23 March to 1 July 2020, with clinical diagnoses of probable COVID-19 were initially included in the study. The 336 patients were searched for in an IMSS database denominated ‘online notification system for epidemiological surveillance’ (Sistema de notificación en línea para la vigilancia epidemiológica, SINOLAVE) by the IMSS. SINOLAVE includes the demographic and clinical data of patients, as well as the diagnoses of SARS-CoV-2 obtained through viral nucleic acid detection by real-time reverse transcription–polymerase chain reaction (RT-PCR) assays. These data are notified by The State Laboratory of Public Health from the Secretary of Health of Michoacán and endorsed by the Institute of Diagnosis and Epidemiological Reference. A total of 31 patients were not found in the SINOLAVE database and, thus, were eliminated in the first step. A further 96 SARS-CoV-2-negative patients were removed from the study. Of the 209 SARS-CoV-2-positive patients, eight with unknown outcomes regarding recovery or death were removed. Finally, 82 patients were removed because, when reviewing each of their records, it was observed that the WBC counts were performed while they were administered steroids. Therefore, the final population included in this study comprised 119 hospitalized patients (see the Figure 1). 

A total of 278 WBC counts with their respective WBC differential counts were analyzed. The WBC counts from the 119 hospitalized patients were obtained in 1 to 29 days—from the onset of clinical symptoms until they were discharged or administered corticosteroids—providing a total of 152 WBC counts from the 59 recovered patients and 126 WBC counts from the 60 deceased patients (Figure 1). Thus, most of the patients had more than one WBC count available over the course of the disease. No patient had more than one WBC count on the same day. As systemic and inhaled corticosteroids modify the number of circulating leukocytes, which then return to normal 24–48 h after administration [31,32,33,34], WBC counts were eliminated if they were performed less than 48 h after the last administration of any type of inhaled or systemic corticosteroid.

### 3.2. Demographics, Clinical Characteristics, and Diagnosis

The numbers of community-infected patients who recovered and died were 59 (49.6%) and 60 (50.4%), respectively. The mean number of days from hospital admission to discharge for the recovered patients was 13.54 ± 7.3, and that for the deceased patients was 13.36 ± 7.7. The minimum number of days for which patients in the recovered group remained hospitalized was 4 days, while that for the deceased group was 3 days. However, the maximum number of days that one patient with the outcome of death remained hospitalized was greater (57 days) than that of a patient in the recovered group (38 days). 

Most of the recovered patients ranged from 27 to 49 years in age, while most of the deceased patients ranged from 60 to 90 years in age (Table 1). Furthermore, the estimated mean survival time after admission was lower in patients aged 60 to 90 years old (15.9 days; SE ± 1.4; 95% CI) than in patients aged 26 to 59 years old (27.8 days; SE ± 4.0; 95% CI; *p* = 0.002). Most of the patients were male (*p* < 0.0001), with no statistically significant difference between the recovered and deceased males. Most of the recovered patients aged 27 to 49 years were male (Table 1). 

Half of the patients did not present any comorbidity; however, among the rest of the patients, the most frequent comorbidities were hypertension and diabetes. There was a higher proportion of hypertension in the group of deceased patients, in comparison with the group of recovered patients, with a statistically significant difference between groups (Table 1). However, the estimated survival time was 27.0 days (SE ± 3.9; 95% CI) after admission to the hospital in patients without hypertension, and 16.9 days (SE ± 1.9; 95% CI) in those with hypertension (*p* = 0.027). The proportions of diabetic patients were almost equivalent in both groups (Table 1). 

The most common signs and symptoms experienced upon admission in patients were fever, dry cough, headache, myalgia, arthralgia, and dyspnea. The less common signs and symptoms were dysgeusia, anosmia, and coryza. Furthermore, a lower number of patients were unconscious when admitted to the hospital. There were no statistically significant differences in any of the signs and symptoms between the groups of recovered and deceased patients (Table 1). 

Upon admission, most of the patients in the two groups and overall were diagnosed with severe acute respiratory infection (SARI). However, there were more intubated patients with the outcome of death. With respect to medication, the deceased patients received more antibiotics and oseltamivir than the patients who recovered. Most of the patients received corticosteroids during their hospital stays, with no difference between the groups. The deceased patients received more hydrocortisone than and two different corticosteroids from the recovered patients (Table 1).

### 3.3. Comparison of NLRs and WBC Differential Counts of COVID-19 Patients with Different Outcomes

**Figure 2:**
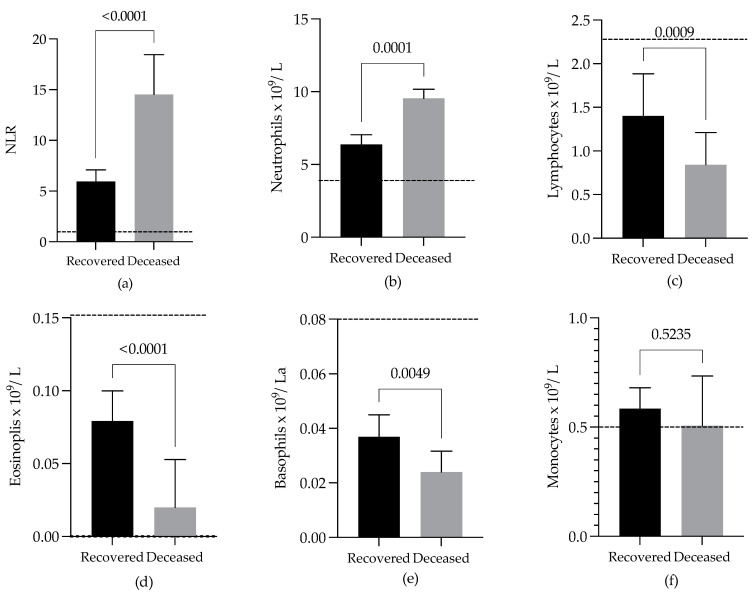
Comparison of (**a**) neutrophil-to-lymphocyte ratios (NLRs) and (**b**–**f**) white blood cell WBC differential counts of COVID-19 patients with different outcomes. The median values of 152 WBC differential counts with their respective NLRs from 59 recovered patients were compared with the median values of 126 WBC differential counts and NLRs from 60 deceased patients. Bars indicate standard errors. The dashed lines indicate the means of normal values of NLRs or leukocytes. The analysis was performed using the Wilcoxon matched-pairs test.

Our data demonstrate that the WBC differential counts and the NLRs were notably altered in COVID-19 patients, compared with the values reported for healthy subjects of the Mexican population taken as a reference [29] (see Table 2). The mean WBC count of the recovered patients increased slightly (8.57 × 10^9^/L SD ± 3.61) and was 1.5 times higher than that of the deceased patients (10.81 × 10^9^/L SD ± 3.80), compared to the mean normal values (Table 2), with a significant difference between the groups (*p* = 0.0006). The mean NLR for the recovered patients was 2.5 times less (6.10 SD ± 4.60) than that observed for the deceased patients (15.00 SD ± 11.00) and was more than three times that reported for healthy subjects (Table 2). The difference in the medians between the recovered and deceased patients was statistically significant (Figure 2a).

The mean neutrophil count was above the normal values in the recovered patients (6.5 × 10^9^/L SD ± 3.3), with a greater increase in the deceased patients (9.2 × 10^9^/L SD ± 3.6), and a significant difference between the two groups was observed (Table 2; Figure 2b).

The mean lymphocyte count was considerably reduced in the recovered (1.30 × 10^9^/L SD ± 0.67) and deceased patients (0.90 × 10^9^/L SD ± 0.66), compared with the normal values (Table 2). The median lymphocyte count was statistically significantly higher in the recovered patients than in the deceased patients (Figure 2c).

In the recovered patients, the mean absolute number of circulating eosinophils was slightly reduced (0.07 × 10^9^/L SD ± 0.07; 2 times), while in the deceased patients, this reduction was stronger (0.03 × 10^9^/L SD ± 0.07; 5 times), when compared with the normal values (Table 2). The median eosinophil count in the recovered patients was significantly higher than the median in the deceased patients (Figure 2d). The recovered patients registered eosinophil counts of zero in one or more of their WBC differential counts in a lower proportion (49.2%) than the deceased patients (78.3%), a result that was significant (*p* = 0.0012).

The recovered and deceased patients showed marked decreases in basophil counts (0.03 SD ± 0.03 and 0.02 SD ± 0.02 × 10^9^/L) with respect to the mean normal values (Table 2). There was a significant difference between the groups (Figure 2e). 

The mean monocyte count remained very close to the normal values (Table 2) in the recovered and deceased patients (0.57 SD ± 0.26 and 0.53 SD ± 0.36 × 10^9^/L), with no difference between the groups (Figure 2f). Therefore, in contrast to the monocyte counts, the NLR values and neutrophil, lymphocyte, eosinophil, and basophil counts were significantly different between the groups of patients (Figure 2a–f). The eosinophil counts showed the greatest difference between the recovered and deceased patients compared to the other types of leukocytes (Figure 2b–f).

Furthermore, the analysis using a paired parametric test showed differences in the NLR values (*p* < 0.0001) and total leukocyte (*p* = 0.0113), neutrophil (*p* = 0.0047), lymphocyte (*p* = 0.0011), eosinophil (*p* < 0.0001), and basophil (*p* = 0.0068) counts between the groups of patients, the difference in eosinophil counts being greater. In this test, the monocyte counts did not differ between the groups (*p* = 0.6102).

### 3.4. Behavior of NLRs and WBC Differential Counts in COVID-19 Patients with Outcomes of Recovery and Death over the Course of the Disease

We recorded the WBC counts for recovered patients from Days 1 to 29 after the onset of clinical symptoms. On Day 1 and from Days 24 to 29, there were WBC counts from a single patient, while there were records for deceased patients from Days 1 to 28. On Day 22 and from Days 24 to 28, there were WBC counts from a single patient (Table S1). On average, the increase in total leukocytes remained lower than double, with respect to the normal values, in both groups of patients during the course of the disease (Table S1). 

**Figure 3:**
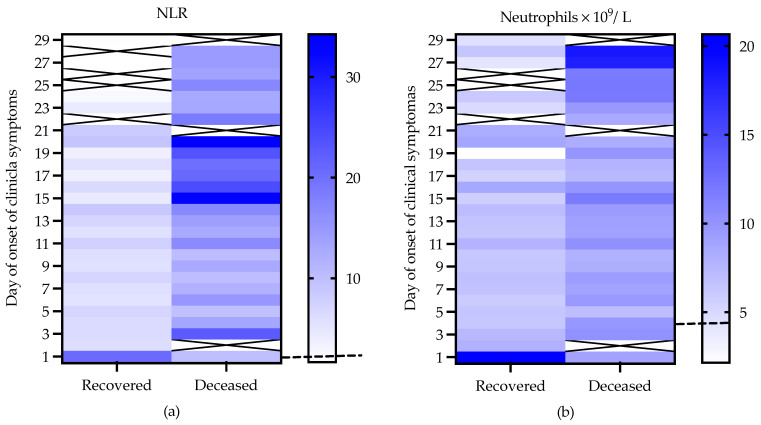

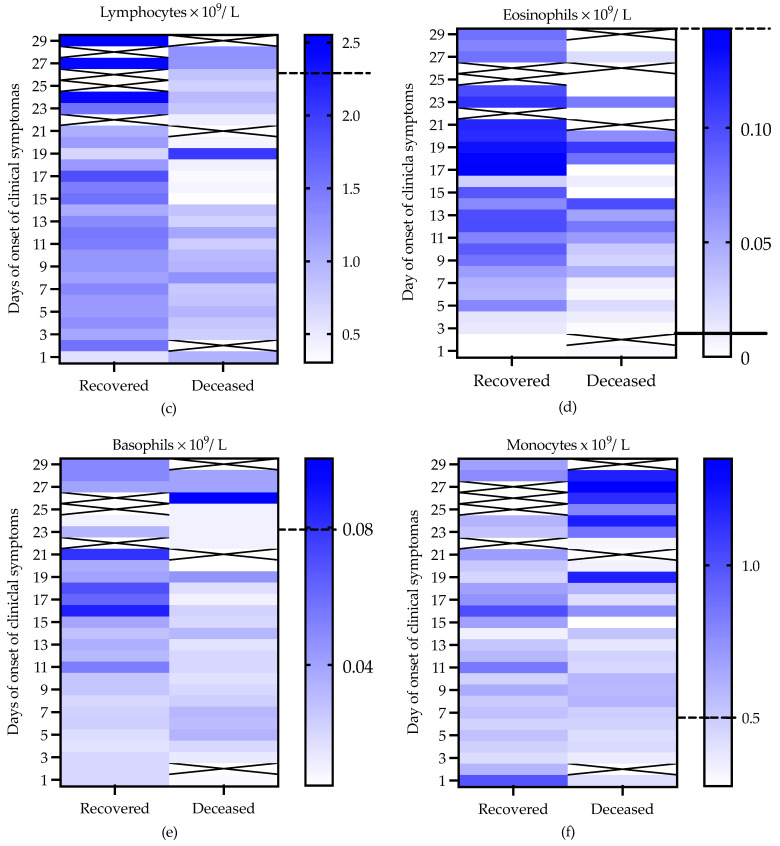
Behavior of (**a**) neutrophil-to-lymphocyte ratios (NLRs) and (**b**–**f**) white blood cell (WBC) differential counts in COVID-19 patients with different outcomes over the course of the disease. The dashed lines indicate the means of normal values of NLR or leukocytes. The continuous line in (**d**) delimits the value for eosinopenia.

From Days 2 to 23, the recovered patients exhibited an increase in NLR of two to four times, with respect to normal values; meanwhile, the deceased patients showed an increase in NLR of five to eleven times, with respect to normal values, over the course of the disease. The highest values were registered on Day 3 and from Days 15 to 23 (Table S1; Figure 3a).

The recovered patients showed neutrophil counts elevated 1.5–2 times, with respect to the normal values, over the course of the disease, while the deceased patients showed increases little more than twice over the course of the disease, although an increase in the neutrophil count of more than four times from Days 27 to 28 was registered for a single patient (Table S1; Figure 3b).

The recovered patients presented lymphocyte counts of approximately half the normal values up to Day 23. One recovered patient presented normal values from Day 24. In the deceased patients, the lymphocyte count values remained from very low to normal from the first day until the last day registered (Table S1; Figure 3c). 

**Figure 4:**
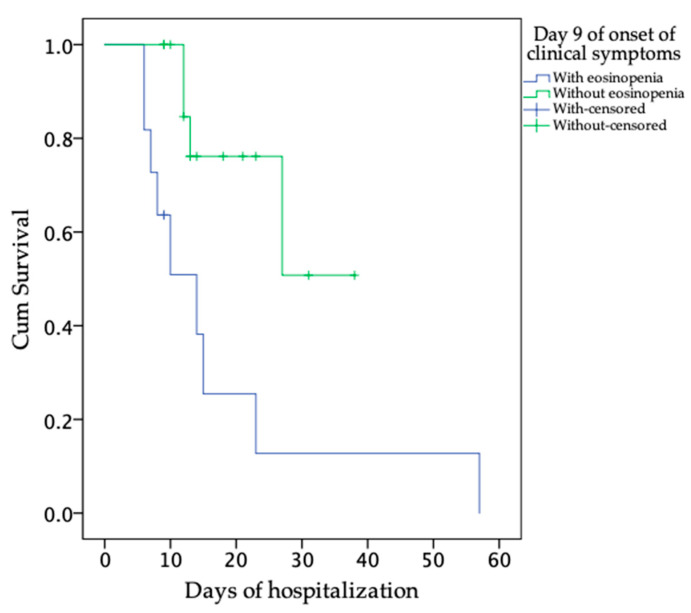
Kaplan–Meier survival function from the time of admission to the time of death in COVID-19 patients with eosinopenia (eosinophil counts < 0.01 × 10^9^/L) and without eosinopenia at Day 9 of onset of clinical symptoms. The probability of survival was within a period of 3 to 57 Days—that is, the period in which the cohort of COVID-19 patients analyzed remained hospitalized. Recovered patients (*n* = 15); deceased patients (*n* = 14).

**Figure 5:**
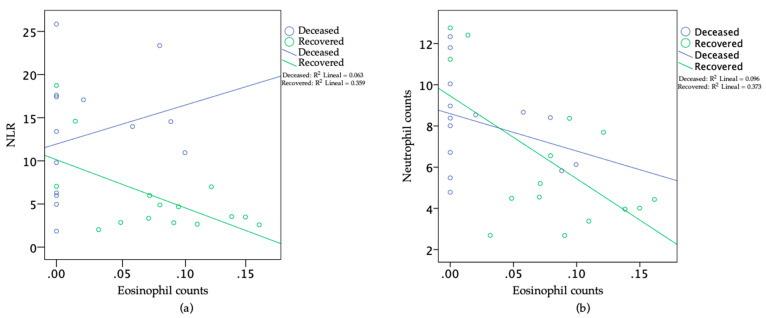
Scatter plots of eosinophil counts (×10^9^/L) against (**a**) the neutrophil-to-lymphocyte ratio (NLR) and (**b**) the neutrophil counts (×10^9^/L) at Day 9 of onset of clinical symptoms for recovered patients (*n* = 15) and deceased patients (*n* = 14). Each NLR value or eosinophil/neutrophil count corresponds to a single patient.

In the recovered patients, we found eosinophil values from 0 to 0.1 × 10^9^/L until Day 16. From Day 17, the eosinophil counts increased to values higher than 0.1 × 10^9^/L and remained at this level until Day 24. One recovered patient showed values below 0.1 × 10^9^/L from Days 24 to 29. On the other hand, in most of the deceased patients, the eosinophil counts remained at values associated with eosinopenia (eosinophil counts < 0.01 × 10^9^/L) or values < 0.1 × 10^9^/L over the course of the disease (Table S1; Figure 3d). Importantly, the estimated mean days of survival after admission (with 95% CI) were greater in patients without eosinopenia than in those with eosinopenia at Day 9 (29.09; SE ± 5.49; 17.60; SE ± 5.80; *p* = 0.007) after the onset of post-clinical symptoms, with a period of 3 to 57 days of hospitalization (Figure 4). Furthermore, on Day 9, we found negative correlations (*p* ≤ 0.0220) of the eosinophil counts with the NLRs (Pearson correlation = 0.60) and with the neutrophil counts (Pearson correlation = 0.62) in the recovered patients (*n* = 15) but not in the deceased patients (*n* = 14); see Figure 5a,b.

Over the course of the disease, most of the recovered and deceased patients showed basophil counts below the values established for healthy patients, which was more pronounced in the deceased patients (Table S1; Figure 3e).

Similar to the NLR and neutrophil count, the monocyte count measured on the first day in the recovered patients was higher (0.99 × 10^9^/L) than the normal values. At Day 2, the values were close to normal in most of the recovered patients and remained roughly constant until Day 10. With the exception of Day 16, on which the monocyte count was observed to be around twice the normal values, the monocyte counts of the recovered patients from Days 11 to 29 were slightly higher than the normal values. The monocyte counts in the deceased patients were below the normal values from Days 1 to 4, remained close to the normal values from Days 5 to 18, and increased from Days 19 to 28, reaching around twice the normal values (Table S1; Figure 3f).

## 4. Discussion

The first case of an imported SARS-CoV-2 infection (from Italy) was detected in Mexico City on February 27 [35]. On March 21, the first imported infections (from Spain) were detected in Morelia, Michoacán, the place of the study [36]. The period of this study was from March 23 to July 1, with no imported cases, so the analyzed cases of COVID-19 were the first community-acquired cases in this province of Mexico. 

As indicated in other studies of COVID-19 patients from Mexico [37,38], we also observed that patients older than 60 years and patients with hypertension were more vulnerable to mortality. However, with respect to obesity and diabetes, there was no statistically significant difference between groups (Table 1). This may be due to the fact that the number of hospitalized COVID-19 patients in this study was small (*n* = 119) compared to the number of patients included in other studies (i.e., *n* = 19,831 and 95,458) [37,38].

A European study that included 2581 ambulatory and hospitalized COVID-19 patients reported that olfactory dysfunction was more prevalent in mild patients (85.9%) than individuals presenting moderate-to-critical disease (4.5–6.9%) [39]. Considering that 84.1% of the patients included in this study were afflicted with a severe-to-critical acute respiratory infection, characterized by the presence of fever, dry cough, and dyspnea, our data were similar to those of this report, in that only 1.7% of the total patients presented anosmia (Table 1).

A systematic review that included three studies with a total of 294 COVID-19 patients, 75 (25.5%) of whom presented severe illness, reported that the eosinophil counts were not significantly different between patients with or without severe COVID-19, and concluded that eosinopenia may not be associated with the unfavorable progression of COVID-19 [28]. However, in this study, the authors did not consider whether the patients had recovered or died. By contrast, a retrospective observational study with a total of 85 fatal cases with clinically diagnosed COVID-19 showed that, upon admission, the absolute eosinophil count in the peripheral blood was reduced in almost all the patients who died (81.2%). These data led the authors to conclude that eosinopenia may indicate a poor prognosis. Nevertheless, this cross-sectional study did not include recovered patients [12]. Additionally, it has been speculated that the eosinopenia associated with COVID-19 is probably a secondary phenomenon that does not directly contribute to the course of disease [19]; however, our data suggest that eosinopenia associated with COVID-19 contributes to the final outcome of the disease. Specifically, the analysis of our data suggested that, in COVID-19 patients, eosinopenia has a negative effect, while an increase in eosinophils has a protective effect. These assertions are based on:

(1) The fact that, on average, the eosinophil counts were higher in the recovered than in the deceased patients (Figure 2d);
(2) Although both groups of patients presented eosinophil count values of zero in one or more of their WBC differential counts, this situation was more frequently observed in the deceased than in the recovered patients. Furthermore, on average, the eosinophil counts in the recovered patients increased faster, up to values greater than 0.1 × 10^9^/L, than those in deceased patients over the course of the disease (Figure 3d);
(3) The estimated mean days of survival after admission was greater in the patients without eosinopenia at Day 9 of the onset of clinical symptoms than in the patients with eosinopenia at the same time (Figure 4);
(4) As COVID-19 progresses, an elevated number of circulating neutrophils and elevated level of NLR have been shown to be an indicator of the severity of respiratory symptoms and poor clinical outcomes [4,40]. In this study, the recovered patients, but not deceased patients, showed high negative correlations between the eosinophil and neutrophil counts, as well as between the eosinophil counts and the NLR, at Day 9 of the onset of clinical symptoms (Figure 5a,b).

It has been suggested that eosinophilia plays a protective role in COVID-19 patients. A controlled study with 314 confirmed symptomatic cases of COVID-19 reported that patients with eosinophilia had lower levels of CRP, milder clinical courses, and better disease outcomes than those without eosinophilia [41]. Another report has revealed that COVID-19 patients (*n* = 10) may benefit from sustained lopinavir treatment and that an increasing eosinophil number may serve as an indicator of COVID-19 improvement [25]. Furthermore, in a retrospective study with 78 patients admitted to an intensive care unit for acute respiratory failure related to SARS-CoV-2 pneumonia, 33% of patients developed eosinophilia; this condition was associated with decreased mortality [42]. Retrospective studies of COVID-19 patients, including patients with diagnoses of asthma, concluded that eosinophilia—in both those with and without asthma—may be associated with a reduced mortality risk [43,44]. These studies agree with the beneficial role of eosinophilia observed in our study. Our data indicate that, as eosinophils increased, the inflammatory cell markers (NLRs and neutrophils) decreased, and vice versa, suggesting that the increase in eosinophils in the recovered patients protected against the exacerbation of inflammation, protection that was lacking in the deceased patients. Importantly, in a prospective study involving 80 COVID-19 patients, it was found that high eosinophil numbers were associated with reduced anticoagulant effect of low molecular weight heparin. However, 95.2% of the study group did not show complications, and the general conditions of the patients did not deteriorate [45]. Additionally, retrospective case series have suggested that eosinophilia and exanthemas may result from an interaction between the antiviral immune response and drugs such as tocilizumab, hydroxychloroquine, and lopinavir/ritonavir [46,47,48,49]. 

Several clinical studies have reported eosinopenia, lymphopenia, and neutrophilia in a high proportion of patients with confirmed SARS-CoV-2 infection, regardless of outcome [3,4,12,13,14,15,16,17,40,50]. Studies considering hematological and immunological variations associated with the outcomes of COVID-19 patients from China have shown that the counts of lymphocytes and eosinophils decreased markedly, while increases in neutrophil counts and NLRs were predominant in fatal patients [4,51]; however, to our knowledge, this is the first study to report that survival during the hospitalization period in COVID-19 patients who did not have eosinopenia at Day 9 after the onset of clinical symptoms was higher than that in patients with eosinopenia, as well as showing that, after the onset of clinical symptoms (Day 9), in recovered patients but not in deceased patients, as the eosinophil count increased, the NLR and the neutrophil count decreased. The latter indicates that eosinophils may indirectly contribute to the control of neutrophil-induced inflammation, preventing the consequences of exacerbated inflammation in those patients who recovered. This makes sense because, although the role of eosinophils in inflammation remains controversial, numerous studies in animal models and in humans have collectively indicated that eosinophils have several different functional roles, exerting proinflammatory, inhibitory, and/or regulatory effects at inflamed sites due, in part, to the existence of distinct eosinophil subpopulations in the same tissue [52]. Specifically, regarding the role of eosinophils in resolving inflammation, it has been observed in murines that eosinophils are recruited to the inflamed loci during the resolution phase, where they locally produce anti-inflammatory and pro-resolving lipid mediators, such as protectin D1 and resolvin E3, through a 12/15-lipooxygenase-mediated biosynthetic route. This promotes resolution by counter-regulating the neutrophil influx and stimulating the ingestion of apoptotic neutrophils by macrophages, as well as increasing phagocyte clearance into draining lymph nodes [18,53,54]. IL-5 is the key driver of eosinophilic differentiation and survival [55]. Thus, this study indicates that contrary to what has recently been suggested [56], hospitalized COVID-19 patients at risk of fatal outcomes should not be treated with anti-IL-5 drugs, such as mepolizumab.

Our study had some limitations. For example, it was not possible to obtain data on the behavior of cytokines and other inflammatory mediators or on leukocyte subsets. Furthermore, the number of patients and WBC counts included was relatively small; as such, it was difficult to properly evaluate the probability of survival in patients hospitalized with eosinopenia and the correlations between the eosinophil counts and the counts of the other leukocytes. 

## 5. Conclusions

Overall, our data analysis suggested that the detection of eosinopenia at Day 9 in COVID-19 patients might serve to predict a poor prognosis for the disease and that eosinophils have a beneficial function in COVID-19 patients, probably through contributing to the control of the exacerbated inflammation induced by neutrophils, which can influence the outcome of the disease.

## Figures and Tables

**Table 1:** Demographics, baseline characteristics, and diagnoses of the patients, classified according to the outcome.

	Recovered	Deceased	Total	*p* (Recovered vs. Deceased)
	*n* = 59	*n* = 60	*n* = 119	
**Age groups (year)**				
20–26	2 (3.4%)	0 (0%)	2 (1.7%)	>0.05
27–49	∗ 25 (42.4%)	8 (13.3%)	33 (27.7%)	<0.005 ∗ 0.0115
50–59	15 (25.4%)	21 (35%)	36 (30.3%)	>0.05
60–90	17 (28.8%)	31 (51.7%)	48 (40.3%)	0.0076
**Sex**				
Female	21 (35.6%)	19 (31.7%)	40 (33.6%)	>0.05
Male	∗ 38 (64.4%)	41 (68.3%)	79 (66.4%)	>0.05
**Comorbidities**				
Any	30 (50.8%)	32 (53.3%)	62 (52.1%)	>0.05
COPD	5 (8.5%)	3 (5%)	8 (6.7%)	>0.05
Smoking	2 (3.4%)	5 (8.3%)	7 (5.9%)	>0.05
Asthma	1 (1.7%)	2 (3.3%)	3 (2.5%)	>0.05
Obesity	6 (10.2%)	7 (11.7%)	13 (10.9%)	>0.05
Cardiovascular disease	1 (0.2%)	4 (6.7%)	5 (4.2%)	>0.05
Hypertension	16 (27.1%)	27 (45%)	43 (36.1%)	0.0304
Diabetes	17 (28.8%)	20 (33.3%)	37 (31.0%)	>0.05
Immunosuppression	1 (1.7%)	1 (1.7%)	2 (1.7%)	>0.05
Renal disease	2 (3.4%)	3 (5%)	5 (4.2%)	>0.05
Cancer (remission)	1 (0.2%)	0 (0%)	1 (0.8%)	>0.05
Tuberculosis	0 (0%)	1 (1.7%)	1 (0.8%)	>0.05
**Signs and symptoms upon admission**				
Sudden start	9 (15.3%)	10 (16.7%)	19 (16.0%)	>0.05
Attack on the general state	27 (45.7%)	31 (51.7%)	58 (48.7%)	>0.05
Fever	50 (84.7%)	54 (90%)	104 (87.4%)	>0.05
Dry cough	51 (86.4%)	55 (91.7%)	101 (84.9%)	>0.05
Headache	48 (81.4%)	42 (70%)	100 (84.0%)	>0.05
Odynophagia	20 (33.9%)	19 (31.7%)	39 (32.8%)	>0.05
Myalgia	39 (66.1%)	41 (68.3%)	80 (67.2%)	>0.05
Arthralgia	38 (64.4%)	37 (61.7%)	75 (63.0%)	>0.05
Prostration	5 (8.5%)	6 (10%)	11 (9.2%)	>0.05
Rhinorrhea	9 (15.3%)	9 (15%)	18 (15.1%)	>0.05
Chills	15 (25.4%)	20 (33.3%)	35 (29.4%)	>0.05
Abdominal pain	4 (6.8%)	6 (10%)	10 (8.4%)	>0.05
Chest pain	31 (52.5%)	32 (53.3%)	63 (53%)	>0.05
Dyspnea	53 (89.8%)	50 (83.3%)	103 (86.6%)	>0.05
Dysgeusia	1 (1.7%)	5 (8.3%	6 (5.0%)	>0.05
Anosmia	0 (0%)	2 (3.3%)	2 (1.7%)	>0.05
Conjunctivitis	8 (13.6%)	4 (6.7%)	12 (10.1%)	>0.05
Coryza	1 (1.7%)	0 (0%)	1 (0.8%)	>0.05
Diarrhea	5 (8.5%)	12 (20%)	17 (14.3%)	>0.05
Unconscious	2 (3.4%)	0 (0%)	2 (1.7%)	>0.05
**Diagnosis**				
Influenza-like illness	6 (10.2%)	10 (16.7%)	16 (13.4%)	>0.05
SARI	53 (89.8%)	50 (83.3%)	103 (86.5%)	>0.05
Clinical pneumonia	20 (33.9%)	23 (38.3%)	43 (36.1%)	>0.05
Radiographic pneumonia	15 (25.4%)	17 (28.3%)	32 (26.9%)	>0.05
**Endotracheal intubation**	4 (6.8%)	21 (35.0%)	25 (21.1%)	<0.0002
**Medications**				
Oseltamivir	2 (3.4%)	9 (15%)	11 (9.2%)	>0.05
Antibiotics	8 (13.6%)	18 (30%)	26 (21.8%)	>0.05
Tocilizumab	0 (0%)	2 (3.3%)	2 (1.7%)	>0.05
**Corticosteroids**	32 (54.2 %)	41 (68.3%)	73 (61.3%)	>0.05
Dexamethasone	22 (37.3%)	20 (33%)	41 (34.5%)	>0.05
Methylprednisolone	16 (27.1%)	10 (16.7%)	16 (13.5%)	>0.05
Hydrocortisone	1 (1.6%)	8 (13.3%)	9 (7.6%)	0.0322
Prednisone	2 (3.4%)	5 (8.3%)	7 (5.9%)	>0.05
Inhaled budesonide and fluticasone	1 (1.6%)	5 (8.3%)	6 (5.0%)	>0.05
Two different corticosteroids	1 (1.6%)	13 (21.7%)	14 (11.8%)	0.0010

Variables were analyzed using χ^2^ test or Fisher’s exact test. Age groups were arbitrarily organized. ∗ Comparison between male age groups with significant difference. Chronic obstructive pulmonary disease (COPD); severe acute respiratory infection (SARI). Antibiotics: penicillin, amoxicillin, ceftriaxone, ciprofloxacin, clarithromycin, and clindamycin.

**Table 2:** Descriptive statistics of NLR, WBC counts, and WBC differential counts of all COVID-19 patients analyzed.

	Minimum	Maximum	Std. Error	Mean	Mean for Healthy Subjects ^‡^
WBC counts	3.2	25	0.23	9.6 ± 3.9	7.01 ± 1.45
NLR	1.08	58.88	0.55	10.5 ± 9.10	1.80 ± 0.65
Neutrophils	1.59	24.01	0.22	7.8 ± 3.7	3.96 ± 1.12
Lymphocytes	0.17	4.53	0.04	1.13 ± 0.70	2.31 ± 0.58
Eosinophils	0.0	0.44	0.00	0.06 ± 0.07	0.15 ± 0.13
Basophils	0.00	0.16	0.00	0.03 ± 0.03	0.08 ∗
Monocytes	0.07	2.2	0.02	0.55 ± 0.31	0.51 ± 0.14

The 278 white blood cell (WBC) counts and respective WBC differential counts and neutrophil-to-lymphocyte ratio (NLR) corresponding to 119 COVID-19 patients; ^‡^ Mean ± Std. Deviation of WBC differential counts of 500 Mexican healthy blood donor volunteers [32,33,29]; ∗ Mean basophil count for healthy subjects was obtained, according to values established by Sysmex^®^ [32,33,30].

## Data Availability

The data are not publicly available due to ethical and legal privacy of the patients included in the study.

## Supplementary Materials

The following are available online at https://www.mdpi.com/article/10.3390/v13091675/s1. Table S1: Descriptive statistics of NLRs, WBC counts, and WBC differential counts of all COVID-19 patients analyzed. Table S2: Correlation of eosinophil counts with NLRs, and the rest of the WBC differential counts of all COVID-19 patients analyzed over the course of the disease.
